# Explaining large mortality differences between adjacent counties: a cross-sectional study

**DOI:** 10.1186/s12889-016-3371-8

**Published:** 2016-08-02

**Authors:** M. Schootman, L. Chien, S. Yun, S. L. Pruitt

**Affiliations:** 1 Department of Epidemiology, Saint Louis University College for Public Health and Social Justice, 3545 Lafayette Avenue, St. Louis, MO 63104 USA; 2 Alvin J. Siteman Cancer Center at Barnes-Jewish Hospital and Washington University School of Medicine, St. Louis, MO USA; 3 University of Texas School of Public Health at San Antonio Regional Campus, Department of Biostatistics, San Antonio, TX USA; 4 Missouri Department of Health and Senior Services, Office of Epidemiology, Jefferson City, MO USA; 5 Department of Clinical Sciences, University of Texas Southwestern Medical Center, Dallas, TX USA

**Keywords:** Bayesian analysis, Neighborhood effects, Spatial statistics

## Abstract

**Background:**

Extensive geographic variation in adverse health outcomes exists, but global measures ignore differences between adjacent geographic areas, which often have very different mortality rates. We describe a novel application of advanced spatial analysis to 1) examine the extent of differences in mortality rates between adjacent counties, 2) describe differences in risk factors between adjacent counties, and 3) determine if differences in risk factors account for the differences in mortality rates between adjacent counties.

**Methods:**

We conducted a cross-sectional study in Missouri, USA with 2005–2009 age-adjusted all-cause mortality rate as the outcome and county-level explanatory variables from a 2007 population-based survey. We used a multi-level Gaussian model and a full Bayesian approach to analyze the difference in risk factors relative to the difference in mortality rates between adjacent counties.

**Results:**

The average mean difference in the age-adjusted mortality rate between any two adjacent counties was −3.27 (standard deviation = 95.5) per 100,000 population (maximum = 258.80). Six variables were associated with mortality differences: inability to obtain medical care because of cost (β = 2.6), hospital discharge rate (β = 1.03), prevalence of fair/poor health (β = 2.93), and hypertension (β = 4.75) and poverty prevalence (β = 6.08).

**Conclusions:**

Examining differences in mortality rates and associated risk factors between adjacent counties provides additional insight for future interventions to reduce geographic disparities.

## Background

It is well established that health outcomes, including disease incidence, life expectancy, and mortality vary geographically. Numerous studies have described the extent of geographic variation in adverse health outcomes including cardiovascular disease, cancer, and behaviors [1–6]. To document geographic variation of adverse health conditions in the U.S., many studies have examined outcomes aggregated by various geographic units, including administrative units such as counties (e.g., http://www.countyhealthrankings.org/, http://www.cdc.gov/brfss/index.html).

There is an extensive literature dealing with spatial patterns that can broadly be conceptualized as studies focused on spatial interdependence (e.g., cluster detection of elevated rates, spatial interaction) and spatial heterogeneity (e.g., spatial variation of predictors and outcomes [7–10]). There is a wealth of methodologies available to test for these spatial patterns, including Bayesian approaches. To quantify the extent of geographic variation in health across administrative units, public health studies typically resort to using global indicators of variation such as measures of the intraclass correlation coefficient [11–13]. One important limitation of this approach is that such global measures cannot quantify differences between adjacent geographic areas, which often have very different mortality rates. Local indicators of spatial association (e.g., Moran’s I) are available and provide local measures of similarity between each area’s associated value and those of nearly areas. However, Moran’s I is incompatible with multilevel approaches and does not allow for examination of reasons for differences between adjacent counties.

Identification and examination of differences in mortality rates between adjacent areas may facilitate identification of the reasons for such differences and unique opportunities for development and implementation of interventions addressing those reasons [14, 15]. Evidence-based resources exist to implement changes in communities that may affect health outcomes, such as CDC’s Community Health Improvement Navigator (http://www.cdc.gov/chinav/index.html), www.stablecommunities.org, and the US Department of Education Promise Neighborhoods (http://www2.ed.gov/programs/promiseneighborhoods/index.html). Identification of reasons for different health outcomes may provide guidance for policy-makers, including those at local health departments, healthcare organizations, and community members. For example, if differences in mortality rates between adjacent counties are driven by availability of medical care services or disease prevalence, interventions can be targeted, implemented, and evaluated appropriately.

The goal of this study was to describe a novel application of advanced spatial statistical methods to 1) examine the extent of differences in mortality rates between adjacent counties, 2) describe differences in risk factors between adjacent counties, and 3) determine if differences in risk factors are associated with differences in mortality rates between pairs of adjacent counties. We focus on the State of Missouri because of the large overall burden of disease and geographic disparities across counties that exist.

## Methods

We conducted a cross-sectional study in Missouri with combined 2005–2009 age-adjusted all-cause mortality rate as the outcome of interest. This time period was selected to take advantage of the 2007 Missouri County-Level Survey that contains prevalence estimates of chronic diseases, conditions, and risk factors [16]. This is a Behavioral Risk Factor Surveillance System-like survey of approximately 800 adults in each of eight counties with metropolitan statistical areas or large proportions of minority populations, and 400 adults in each of the rest of 107 Missouri counties to produce county-specific prevalence estimates of chronic diseases, conditions, and risk factors. The sampling design of this survey used county as the sampling unit, allowing for county-specific estimates. We selected county-level variables associated with adjusted mortality based on previous studies and the Andersen behavioral model of health services use, consisting of predisposing factors, access to healthcare services, use of health services, health behavior, population need for services, and enabling characteristics [17, 18]. The most recent version of this model was extended to include area-level factors, as was used in this study.

### Risk factors

*Predisposing factors* included area racial distribution operationalized by the percentage of African Americans in each county in the year 2000. Differences in age distribution between counties were not examined as part of risk factors examined since they were taken into account by calculating the age-adjusted mortality rates.

*Access to healthcare services* included preventable hospitalization rate, the percentage of the population without healthcare coverage, and the percentage of the population unable to obtain medical care because of cost. The 2005–2009 preventable hospitalization rate per 100,000 population, defined as diagnoses for which timely and effective outpatient care can help to reduce the risks of hospitalization by either preventing the onset of an illness or condition, controlling an acute episodic illness or managing a chronic disease or condition. This rate was obtained from the Missouri Department of Health and Senior Services’ Missouri Information for Community Assessment (MICA) data. The percentage of the population without healthcare coverage and who was unable to obtain medical care because of cost were obtained from the 2007 Missouri County-level survey.

*Use of health services* included number of visits to emergency departments and number of hospital discharges during 2005–2009, per 100,000 population. Both variables were obtained from MICA.

*Health behavior* among the population aged 18 or older included the percentage who reported being a current smoker and those who reported not having any leisure time physical activity during the past 30 days. Both variables were obtained from the 2007 county-level survey.

Characteristics of the *need* of the county population included the percentage who reported 1) fair or poor health, 2) body-mass index of at least 25.0 based on height and weight, 3) ever being told by a healthcare professional to have high blood pressure, and 4) ever been told by a healthcare professional to have diabetes (not including gestational diabetes). All four characteristics were obtained from the 2007 county-level survey.

*Enabling characteristics* included percentage of the county population living in poverty based on the 2000 U.S. Census.

### Statistical analysis

To determine the extent to which the difference in risk factors could affect the difference in mortality between any pair of adjacent counties, we first defined the county of interest (j) and identified all its neighbors. Neighboring counties share at least part of their boundary (queen-based neighbor), regardless of the length of that border. Each county has at least one neighboring county. When county j and its neighboring counties Θ_j_ = (1, 2, …, i) are defined, the difference in mortality rates between county j and a neighboring county i is calculated as ΔY_ij_ = Y_j_-Y_i_. Similarly, the difference in a risk factor between county j and a neighboring county i is ΔX_ij_ = X_j_-X_i_. Hence, the new data can be organized as a two-level framework with level-1 (neighboring county j) data nested in a level-2 (county i) index. An initial multi-level Gaussian model can be established by:

$$ \Delta {\mathrm{Y}}_{\mathrm{ij}}=\left({\upbeta}_{\mathrm{o}}+{\mathrm{b}}_{\mathrm{o}\mathrm{j}}\right)+{\sum}_{\mathrm{k}=1}^{\mathrm{p}}\left({\upbeta}_{\mathrm{k}}+{\mathrm{b}}_{\mathrm{k}\mathrm{j}}\right)\times {\left(\Delta {\mathrm{X}}_{\mathrm{k}}\right)}_{\mathrm{ij}}+{\mathrm{f}}_{\mathrm{spat}}\left(\mathrm{s}\right), $$

where the fixed coefficient β_k_ can be explained by the average change on the difference of mortality rates ΔY_ij_ by a unit change (i.e., percentage or per 100,000 population) of the difference of the risk factor (ΔX_k_)_ij_ between a county and its neighbors. The random intercept b_0j_ represents the j^th^ county’s deviation from the population mean intercept, and the random slopes b_kj_ imply that the influence of covariates vary from county to county. The last term f_spat_(s) is a structured spatial function estimated by the Markov random fields (MRF) to control the spatial autocorrelation and to account for the spatial heterogeneity. The MRF has a conditional autoregressive prior with a normal distribution having mean ${\displaystyle {\sum}_{\mathrm{s}\hbox{'}\in {\upomega}_{\mathrm{s}}}{\mathrm{f}}_{\mathrm{s}\mathrm{pat}}\left(\mathrm{s}\hbox{'}\right)}/{\mathrm{N}}_{\mathrm{s}}$ and variance σ_spat_^2^/N_s_, where N_s_ is the number of neighboring counties, and s'∈ω_s_ means that county s' is one of the neighboring counties of county s. All unknown variance parameters of random effects were assigned an inverse Gamma prior IG(0.001, 0.001), and all fixed effects used a diffuse prior. Because the sum of a random effect is fixed at 0, there is no need to specify a prior for the mean parameter of a random effect [19].

This model was fitted by a fully Bayesian approach using Markov Chain Monte Carlo techniques by randomly drawing from the full conditional distributions of blocks of parameters given the rest of parameters and the data. In the procedure of model fitting, 25,000 iterations were carried out, with the first 5,000 samples used as burn in. We stored every 20^th^ sample from the remaining 20,000 samples, giving a final sample of 1,000 for the use of parameter estimations. Corresponding 95 % credible intervals (CI) were calculated based on the posterior distribution of the 1,000 samples. Model assessment was based on the deviance information criterion. Models with lower deviance information criterion values, which imply better models, were selected over models with higher values. MCMC convergence was assessed by sampling trace plots. A sensitivity analysis was performed to check the robustness of estimated parameters of the fixed effects of the multivariable model when the two hyperparameters (a, b) are varied for the prior of variance parameters. We used SAS v9.3 (SAS Institute, Cary, NC) for data management and BayesX 2.1 software package for the spatial analysis [20].

**Fig. 1:**
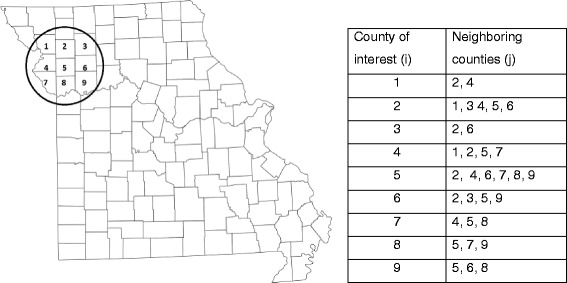
Example of a data structure based on adjacency of counties, which share any part of county boundaries

The data structure based on county adjacency results in lack of data independence because each county can be a neighbor to more than one index county (Fig. 1). For example, if the index county is county 1 (j = 1) then 2 counties are adjacent (i = 2, 4). However, the data become inversed (e.g. *a* and -*a*) when the index county is county 2 (j = 2) and county 1 is again adjacent. We included a modifier variable in the models wherein 1 represents data obtained from adjacent counties relative to each index county, and 0 represents the inverse data.

## Results

**Fig. 2:**
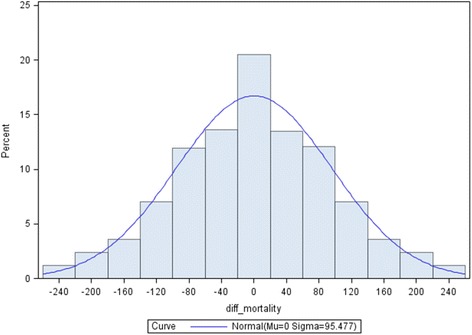
Histogram plot of differences in age-adjusted mortality rates between adjacent counties, Missouri, 2005-2009

**Fig. 3:**
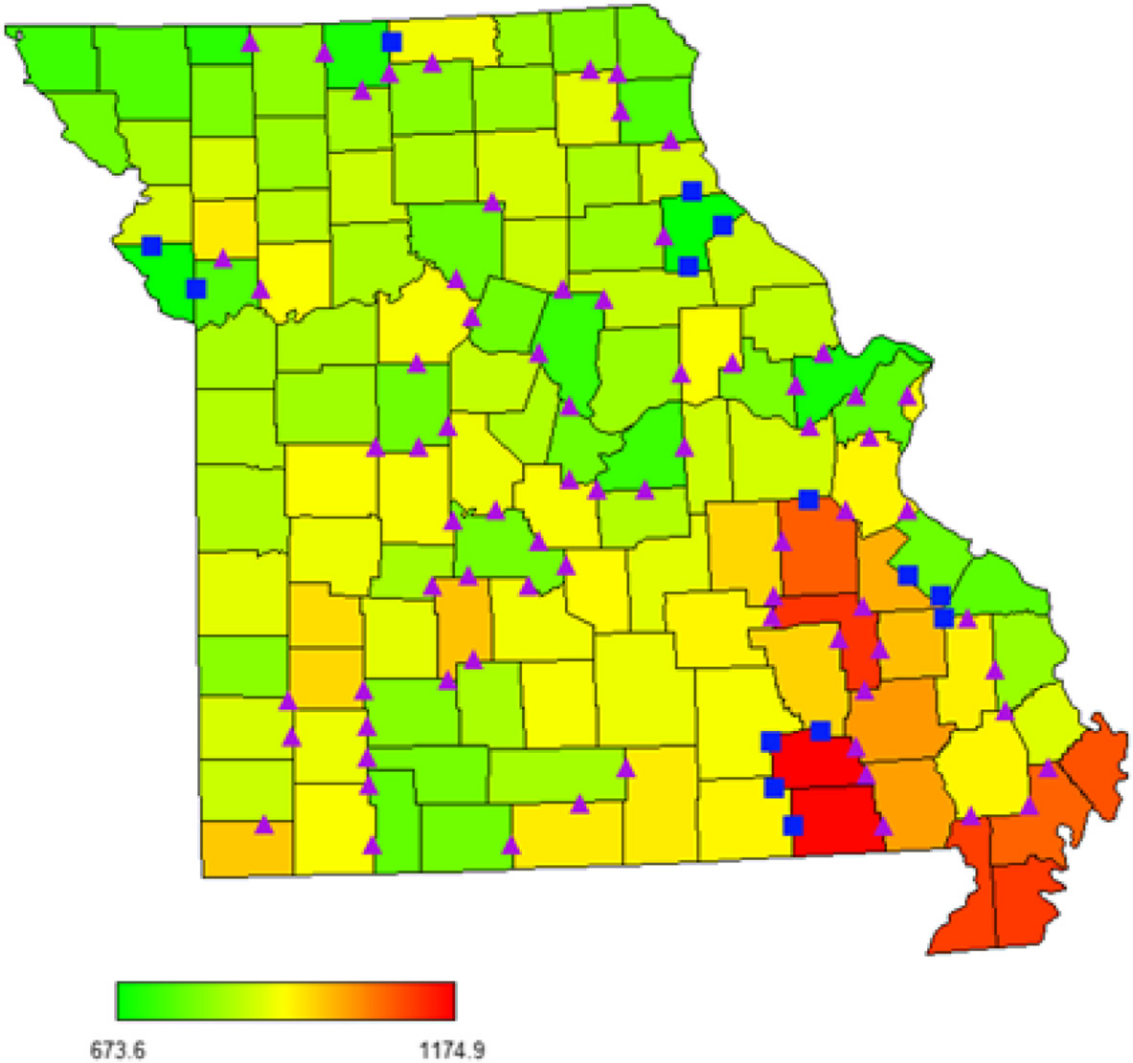
Map of age-adjusted county mortality rates (per 100,000 population), Missouri 2005-2009. *Squares* represent the difference of mortality rates between adjacent counties ≥ twice its standard deviation. *Triangles* represent the difference of mortality rates between adjacent counties ≥ its standard deviation and < twice of its standard deviation

During 2005–2009, there were 219,014 deaths in Missouri for an overall age-adjusted mortality of 853.3 (95 % CI: 849.7, 856.9) per 100,000 population. Age-adjusted mortality rates varied across all 115 Missouri counties from a low of 673.6 to a high of 1,174.9 per 100,000 population (Figs. 2 and 3). The 115 Missouri counties had an average of 5.1 adjacent counties (range = 1 to 8). Number of deaths ranged from 115 to 36,957 across counties. The average mean difference of age-adjusted mortality rates between two adjacent counties was −3.27 (standard deviation [SD] = 95.5) per 100,000 population, with an absolute maximum (|max|) of 258.80 per 100,000 population. Figure 3 also shows differences in adjacent mortality rates that are more than twice the standard deviation of age-adjusted mortality differences (squares). There are several counties in the north-central, western, eastern, and south western parts of Missouri where differences between adjacent counties are large.

Table 1 shows large differences between adjacent counties for some risk factors but small differences for others. For example, while absolute average differences between adjacent counties in percentage of the population who was African American (predisposing factor) was very small (−0.61 %), the absolute difference between some adjacent counties was as large as 49.90 % (SD = 5.73 %). Among access and health services variables, differences between adjacent counties were larger for the hospitalization discharge rate (SD = 25.55; |max| = 101.27; |mean| = 18.56), than for differences in preventable hospitalization rate (SD = 6.89; |max| = 47.77; |mean| = 4.52). For the behavior and need variables, differences between adjacent counties were similar for the prevalence of smoking (SD = 6.09; |max| = 21.3; |mean| = 4.86), lack of physical activity prevalence (SD = 5.60; |max| = 15.10; |mean| = 4.50), fair-poor self-rated health (SD = 4.91; |max| = 18.40; |mean| = 3.78), overweight and obesity prevalence (SD = 5.20; |max| = 17.80; |mean| = 4.09), and hypertension (SD = 3.99; |max| = 11.90; |mean| = 3.18), which were larger than differences diabetes (SD = 2.47; |max| = 6.60; |mean| = 1.98). The difference in poverty rate (enabling variable) between some adjacent counties was large (up to 15.95 %), although the standard deviation of the difference was 4.15 %.

**Table 1:** Data sources and descriptive statistics of differences between adjacent counties

Variable^a^	Data source	Mean	SD	Absolute maximum	Absolute mean
ΔAge-adjusted mortality rate		−3.27	95.50	258.80	75.59
Predisposing factors					
ΔAfrican Americans (%)	Census 2000	−0.61	5.73	49.90	2.81
Access					
ΔPreventable hospitalization rate^b^	MICA 2005-2009	−0.37	6.89	47.77	4.52
ΔPopulation without health insurance (%)	MCLS 2007	−0.25	7.21	23.90	5.56
ΔPopulation unable to obtain medical care because of cost (%)	MCLS 2007	0.13	3.68	12.00	2.97
Use of health services					
ΔEmergency department visits rate^b^	MICA 2005-2009	−0.04	1.33	4.56	1.07
ΔHospital discharge rate^b^	MICA 2005-2009	0.16	25.55	101.27	18.56
Health behavior					
ΔPopulation currently smoking (%)	MCLS 2007	−0.35	6.09	21.30	4.86
ΔPopulation without leisure-time physical activity (%)	MCLS 2007	−0.31	5.60	15.10	4.50
Need					
ΔPopulation in fair or poor health (%)	MCLS 2007	−0.50	4.91	18.40	3.78
ΔPopulation overweight or obese (%)	MCLS 2007	0.05	5.20	17.80	4.09
ΔPopulation with high blood pressure (%)	MCLS 2007	0.08	3.99	11.90	3.18
ΔPopulation with diabetes (%)	MCLS 2007	0.13	2.47	6.60	1.98
Enabling					
ΔPopulation below federal poverty level (%)	Census 2000	−0.30	4.15	15.95	3.31

Abbreviations: *MCLS* Missouri County-Level Survey, *MICA* Missouri Information for Community Assessment, *SD* standard deviation

^a^Δsignifies the difference between adjacent counties

^b^per 100,000 population

All variables were associated with mortality differences between adjacent counties in univariate models except for the difference in the percentage of the population that is African American (Table 2). Stronger associations with larger mortality differences were found for inability to pay for medical care, emergency department utilization, fair or poor health, hypertension, and poverty rate in univariate models. For each additional ER visit per 100,000 population between adjacent counties, the mortality rate significantly increased 16.96 per 100,000 population (95 % CI: 10.45, 24.14). For every 1 % increase in the difference in hypertension, the mortality difference significantly increased 11.19 deaths per 100,000 population (95 % CI: 9.31, 13.10), which was the strongest association among the need-for-medical-care variables. For every 1 % increase in the difference in poverty rate between adjacent counties, the mortality rate difference significantly increased 11.26 per 100,000 population (95 % CI: 9.50, 13.13).

**Table 2:** Associations between differences in risk factors with differences in age-adjusted mortality rates between adjacent counties

	Univariate models				Multivariable model		
Variable	Estimate	95 % CI		DIC	Estimate	95 % CI	
Predisposing factors							
ΔAfrican Americans (%)	0.54	−1.33	2.27	687.66			
Access							
ΔPreventable hospitalization rate^a^	**6.67**	**4.86**	**8.70**	722.07			
ΔPopulation without health insurance (%)	**3.97**	**2.78**	**5.10**	692.23			
ΔPopulation unable to obtain medical care because of cost (%)	**11.77**	**9.70**	**13.97**	688.89	**2.60**	**0.59**	**4.57**
Use of health services							
ΔEmergency department visits rate^a^	**16.96**	**10.45**	**24.14**	695.67			
ΔHospital discharge rate^a^	**1.87**	**1.43**	**2.34**	725.89	**1.03**	**0.66**	**1.38**
Health behavior							
ΔPopulation currently smoking (%)	**4.78**	**3.15**	**6.26**	710.03			
ΔPopulation without leisure-time physical activity (%)	**6.69**	**4.88**	**8.51**	724.84			
Need							
ΔPopulation in fair or poor health (%)	**10.66**	**9.09**	**12.22**	706.82	**2.93**	**1.26**	**4.59**
ΔPopulation overweight or obese (%)	**3.60**	**1.97**	**5.28**	686.08			
ΔPopulation with high blood pressure (%)	**11.19**	**9.31**	**13.10**	686.56	**4.75**	**2.94**	**6.47**
ΔPopulation with diabetes (%)	**5.61**	**1.97**	**8.93**	685.38			
Enabling							
ΔPopulation below federal poverty level (%)	**11.26**	**9.50**	**13.13**	704.97	**6.08**	**4.38**	**7.97**
Deviance information criteria (DIC)					**768.84**		

Abbreviations: *CI* credible interval, *DIC* deviance information criteria

^a^per 100,000 population ^b^Δ signifies the difference between adjacent counties

Bold font indicates estimates with 95 % CI that do not include the value of zero

Table 2 also displays the best fitting multivariable model of only six variables associated with mortality differences between adjacent counties based on the lowest value of the model deviance information criterion. For a 1 % increase in the difference in inability to pay for medical care, the adjusted difference in mortality rates increased 2.60 per 100,000 population (95 % CI: 0.59, 4.57). For every 1 % increase in the difference in hospital discharge rate, the difference in mortality rates increased 1.03 per 100,000 population (95 % CI: 0.66, 1.38). The prevalence rates of poor-fair health status and hypertension were included in the model and showed similar associations with the difference in mortality rates between adjacent counties. Increasing poverty rate differences was associated with larger differences in mortality rates between adjacent counties.

We deleted the modifier variable that represented the inverse data to examine its effect on our findings. Parameter estimates and confidence intervals were unchanged, suggesting that the dependent data structure did not play a role in our findings. Different hyperparameters for the prior of variance parameter had inconsequential effects on our results and did not change the conclusion of our findings (Table 3). Sampling trace plots revealed that all estimates reached convergence successfully (data not shown).

**Table 3:** Sensitivity analysis in the estimated parameters of fixed effects with different hyperparameters (a, b) in the prior of variance parameters

	a = 0.1, b = 0.1			a = 0.01, b = 0.01			a = 0.001, b = 0.001			a = 0.0001, b = 0.0001		
Variable	Estimate	95 % CI		Estimate	95 % CI		Estimate	95 % CI		Estimate	95 % CI	
Intercept	−1.05	−14.36	10.62	−1.34	−12.54	8.85	−1.06	−12.88	10.05	−1.64	−12.32	8.05
ΔPopulation unable to obtain medical care because of cost	1.93	−1.04	4.86	2.60	0.59	4.57	2.16	−0.17	4.46	2.75	0.80	4.74
ΔHospital discharge rate	1.28	0.33	2.23	1.03	0.66	1.38	1.11	0.60	1.65	0.99	0.68	1.32
ΔPopulation in fair or poor health	1.86	−0.49	4.36	2.93	1.26	4.59	2.59	0.57	4.59	3.14	1.40	4.74
ΔPopulation with high blood pressure	6.08	3.50	8.64	4.75	2.94	6.47	5.22	3.20	7.16	4.57	2.96	6.23
ΔPopulation below federal poverty level	6.65	4.05	9.14	6.09	4.38	7.97	6.18	4.20	8.22	6.08	4.34	7.81

Abbreviations: *CI* credible interval

Δ signifies the difference between adjacent counties

## Discussion

We describe a novel application of advanced spatial analysis related to the practice of epidemiology to 1) examine the extent of differences in mortality rates between adjacent counties, 2) describe differences in risk factors between adjacent counties, and 3) determine if differences in risk factors are associated with differences in mortality rates between pairs of adjacent counties. Differences in mortality rates between adjacent counties were large, which provides important information for policymakers on the success of the previously implemented interventions. This may facilitate identification of the reasons for such differences and unique opportunities for development and implementation of interventions [14, 15]. This is even more salient with the Patient Protection and Affordable Care Act (Public Law 111–148, 2010), which may provide the impetus for reducing health disparities between geographic areas. The law is designed to address long-standing racial and socioeconomic inequalities by improving access to quality health care for all Americans through expansion of state Medicaid programs and health insurance exchange subsidies. The Affordable Care Act also removes cost as a barrier to preventive health services, including cancer screening, tobacco dependence counseling and treatment, and obesity screening and counseling.

This also provides evidence that interventions in some counties can be improved. While the statewide age-adjusted mortality was 853.3 per 100,000, differences between adjacent counties were as large as 258.8 deaths per 100,000. This variation is masked when using measures of the intraclass correlation coefficient or other measures typically used in medical geography or spatial epidemiology (e.g., Median Odds ratio), which provide a global indication of the extent of the variation across the entire geographic area but do not have a focus on differences between adjacent areas. We recognize availability of local indicators of spatial associations such as Moran’s I and geographically weighted regression [21]. However, while useful in other studies, local spatial measures such as these were not compatible with our multilevel design. Our novel strategy provides new data suggesting that examination of mortality rates between adjacent counties provides additional information beyond studies that focuses solely on the extent of global variation across geographic areas. Even though mortality rates were high in southeast Missouri, differences between some adjacent counties were not large suggesting that our method to focus on adjacent differences provides additional information beyond simply focusing on county mortality rates.

Differences in risk factors between adjacent counties were also large in some instances. For example, differences in the percentage of the population that is uninsured between adjacent counties were as large as 23.90 %. Larger differences existed in mortality rates between adjacent counties that were associated with differences in access to care (inability to afford medical care), use of health care (hospitalization), the need for health care (health status, hypertension, diabetes), and enabling factors (poverty rate). Our results show that for some variables, the difference in risk factors was not very large between adjacent counties, but their effects on mortality differences were substantial. For example, the maximum difference between adjacent counties was only about seven percent for diabetes, but mortality differences increased by 5.61 deaths per 100,000 population for every 1 % difference in such prevalence. This suggests that interventions that are able to reduce differences in diabetes prevalence between adjacent counties by a relatively small amount also may result in a sizeable reduction in mortality differences. In contrast, variation in hypertension prevalence between adjacent counties was much larger than for diabetes prevalence, and the association with differences in mortality smaller. This suggests that reducing differences in diabetes prevalence between adjacent counties may have a larger impact on reducing differences in mortality rates than focusing on reducing differences in hypertension prevalence. In this example, we recognize that diabetes and hypertension share some risk factors so focusing on diabetes may also affect hypertension prevalence. Interestingly, neither of the behavioral risk factors (smoking, physical inactivity) was included in the multivariable model, but their effects on mortality difference may be indirect through the four need-for-medical-care variables. Policy makers may use these results and local prevalence estimates of these risk factors to set priorities and allocate resources for risk factor reduction in their counties.

Local policy makers, county health departments, healthcare organizations, and community members, may learn valuable lessons from those who have identified reasons for elevated adverse health outcomes and subsequently implemented and evaluated interventions in adjacent areas to reduce their own burden of disease. Interventions that are implemented or resources that are provided to one county can often be scaled up to included additional, adjacent counties. In Missouri, and many other states, county governments have the authority to implement policy changes. Often, local authorities look to adjacent counties about the extent of possible changes in risk factors or health outcomes in their county and act accordingly to implement policy changes. Our results, especially Fig. 3, provide opportunity for local entities to identify the adjacent county that had substantially lower mortality rate in an attempt to lower their mortality rate through interventions.

Our study uses a novel approach by incorporating a spatial approach within a multilevel context. Recent, Arcaya and colleagues used a spatial multilevel approach at the county level. However, they used a 2-stage hierarchical approach, which analyzed the locational data first, and then incorporated all location-specific parameters into another model to generate an overall parameter representing all locations.

Our study also was subject to some limitations. First, aggregating all data to the county level masks within-county variation. However, a similar statistical approach may be used to examine differences between adjacent intracounty units (e.g., census tracts). Second, results for counties near Missouri’s border may be underestimated because the influence from adjacent counties at the borders of neighboring states was not taken into account. Third, we could not adjust for all possible confounding variables, possibly leading to omitted variable bias.

## Conclusions

In conclusion, our spatial analysis approach for examining differences in mortality rates and associated risk factors between adjacent counties is novel for the practice of risk factor epidemiology. This approach provides additional insight into future intervention implementation to reduce differences between adjacent counties and therefore geographic disparity.
